# Prevalence of depression among adults with sickle cell disease in the southern region of Saudi Arabia

**DOI:** 10.12669/pjms.344.14760

**Published:** 2018

**Authors:** Sultan Saad Alsubaie, Mohammad Abdulrahman Almathami, Ahmad Abouelyazid, Mohammad Mana Alqahtani

**Affiliations:** 1 Dr. Sultan Saad Alsubaie, MD. Department of Psychiatry, Armed Forces Hospital, Southern Region, Khamis Mushayt, Saudi Arabia; 2 Dr. Mohammad Abdulrahman Almathami, MD. Department of Psychiatry, Armed Forces Hospital, Southern Region, Khamis Mushayt, Saudi Arabia; 3 Dr. Ahmad Abouelyazid, MD. Department of Community Medicine, Armed Forces Hospital, Southern Region, Khamis Mushayt, Saudi Arabia; 4 Dr. Mohammad Mana Alqahtan, MD. Department of Psychiatry, Armed Forces Hospital, Southern Region, Khamis Mushayt, Saudi Arabia

**Keywords:** Sickle cell disease, Depression, Prevalence, Southern region, KSA

## Abstract

**Objectives:**

Sickle Cell Disease (SCD) as other chronic medical conditions is commonly complicated by psychiatric symptoms. Saudi SCD patients are usually originally from Eastern and Southwestern Provinces. The main objective of our study was to evaluate the prevalence of depression among adults with SCD in southern region of Saudi Arabia. We also studied the sociodemographic profiles for these individuals.

**Methods:**

We conducted a cross-sectional study among subjects (n=78) in Armed Forces Hospital, Southern Saudi Arabia using an Arabic version of a Hamilton Rating Scale for Depression HAM-D that has received widespread use and have undergone reliability and validity testing. The data were analyzed by SPSS 22 package program. Pearson’s chi-squared test is used to examine the association between the categorical outcome variables A p-value less than 0.05 was considered statistically significant.

**Results:**

Most of the participants were young adults (26.4± 9.2 years), single females not working who are originally from Jizan and Mahayel Aseer, Southern Saudi Arabia. The prevalence of depressive symptoms was 85.9%. When the association between depression in SCD patients and different demographic characters was tested, no significant relation between depression and any factors was discovered.

**Conclusion:**

This study confirms that depression is common in adult patients with SCD as confirmed by previous studies. On the other hand, socio-demographic factors were not significant predictors of depression in SCD patients. Further research is needed to explore the magnitude and impact of this problem at the national level.

## INTRODUCTION

Sickle Cell Disease (SCD) is one of the most important genetic diseases in the world. SCD is an autosomal recessive disorder characterized by production of abnormal hemoglobin S and is associated with high morbidity and mortality. Millions of children have suffered from SCD worldwide.^1^ In 1960s, SCD was described for the first time in Saudi Arabia.^2^ There are two distinct forms of SCD in Saudi Arabia, both are referred to as the Saudi-Indian and the Benin haplotypes.^3^’^4^ Saudi SCD patients from Eastern Province have the Arab-Indian (AI) haplotype whereas patients from Southwestern Province have the most severe type: the African origin HBB (b-globin gene) haplotypes, most commonly Benin.^4^,^5^ Although data about prevalence of SCD in Saudi Arabia is limited, it is a comparatively common genetic disorder in our country with percentage of 145 per 1000 in Eastern region followed by 24 per 1000 in southern region.^4^-^7^

SCD as other chronic medical conditions is commonly complicated by psychiatric symptoms. The prevalence of depressive symptoms in sickle cell patients is high compared to the general African American population.^8^ Depression is another burden that affects SCD patients’ quality of life and results in increased morbidity and mortality.^9^ Although many patients with SCD have depression and their relationship has been investigated by many researchers,^8^,^10^,^11^ some cautions needs to be observed in the findings due to wide variations in designs and consistency of the studies.^9^

Most of the studies that investigate depression among SCD patients were conducted in Western and African countries, except one study was done in Eastern Province of Saudi Arabia.^12^ Thus, the literature on depression and SCD in Arab countries including Saudi Arabia is more limited. The aim of this study was to estimate the current prevalence of depressive symptoms among Saudi adults with SCD in southern region attending Hematology clinic and to describe sociodemographic characteristics associated with SCD patients having depression.

## METHODS

The current study was conducted among adults Saudi Arabian patients with SCD during their clinic visits to the hematology clinic at Armed forces hospital southern region AFHSR, Khamis Mushait, Kingdom of Saudi Arabia KSA, from December 1, 2016 to February 28, 2017. The study design was a cross-sectional study that obtained all required ethical approval from ethical review committee from AFHSR, Khamis Mushait, KSA. The study includes all adults both males and females with sickle cell disease without any episodes of vaso-occlusive crises for at least a month who are originally from southern region, KSA; not abusing any type of substances. The data collected included age, gender, marital status, level of education, occupation and economic status. Participants were required to give written consent to participate in the study. The investigators uphold the fundamental principles regarding research on human subjects: respect for persons, beneficence, and justice. For all data collection activities, informed consent was sought from the eligible participants following full disclosure regarding the study before data collection was done.

Questionnaires were administered to patients during outpatient clinic visits. Patients complaining of pain, discomfort, or appeared sick in the clinic were not given the questionnaires. The measurement tool that was used in the study is the 17-item version of Hamilton Rating Scale for Depression HAM-D that has received widespread use and have undergone reliability and validity testing.^13^ HAM-D is a clinician rated scale aimed at assessing depression severity among patients. Its internal consistency (Cronbach’s alpha) was 0.76, and 0.92.^14^,^15^ The total score is obtained by summing the score of each item, 0–4 (symptom is absent, mild, moderate, or severe) or 0–2 (absent, slight or trivial, clearly present). For the 17-item version, scores can range from 0 to 54. It is accepted by most clinicians that scores between 0 and 7 do not indicate the presence of depression, scores between 8 and 13 indicate mild depression, scores between 14 and 18 indicate moderate depression, scores between 19 and 22 indicate severe depression, and scores over 23 indicate very severe depression. It was validated in Arabic culture.^16^

The predictor variables were depression and demographics. The data were analyzed using SPSS for IBM version 22 software system. Descriptive statistics (mean, Standard Deviation (SD), and percentages) were used to describe the quantitative, categorical, and outcome variables. Pearson’s chi-squared test was used to examine the association between the categorical outcome variables. A p value of <0.05 was used to report the statistical significance and precision of the estimates.

## RESULTS

### Sociodemographic characteristics

The mean age of the 78 study subjects was 26 years, with a higher proportion of females (64.1%) than males (35.9%). There were more single subjects (65.4%) than subjects who were married (32.1%) or divorced or widowed (2.6%). All the subjects were originally from southern region of Saudi Arabia, and most of them were from Jizan and Mahayel Aseer (56.4%), (33.3%) respectively; the rest (10.3%) were from Abha, Khamis Mushayt, Ahad Rufieidah, Almajardah, Bareq and Faifa. Those who hold masters, diploma; and intermediate and secondary educational levels were more numerous (39.7%), (43.6%) than subjects who were illiterate or with primary levels of education (16.6%). There were more unemployed subjects (61.5%) than subjects who were students (37.2%) or working (1.3%). The subjects with average income constituted (56.4%) of the sample whereas (39.7%) had good income and surprisingly (3.8%) had poor income. (Table-I):

**Table-I:** Sociodemographic characters of studied group.

Variables	No. (%)
Age (years) Mean (SD) = 26.4 (9.2)	
***Gender***	
Males	28(35.9)
Females	50(64.1)
***Marital Status***	
Single	51(65.4)
Married	25(32.1%)
Divorced or Widowed	2(2.6%)
***Birthplace and Residency***	
Abha	1(1.3%)
Ahad Rufydah	1(1.3%)
Almajardah	3(3.8%)
Bareq	1(1.3%)
Faifa	1(1.3%)
Jizan	44(56.4%)
Khamis Mushayt	1(1.3%)
Mahayel Aseer	26(33.3%)
***Educational Level***	
Illiterate and Primary	13(16.6%)
Intermediate and Secondary	34(43.6%)
University and Diploma	31(39.7%)
***Monthly Income***	
Good	31(39.7%)
Average	44(56.4%)
Poor	3(3.8%)
***Occupation***	
Student	29(37.2%)
Working	1(1.3%)
Not working	48(61.5%)

### Total Depressed and Non- Depressed Score and Prevalence of Depression

Most individuals had depression score equal or above than eight, 85.9% who differ in severity from (mild 28.2%, moderate 21.8%, severe 21.8%, and very severe 14.11%); on the other hand, about 14.1%% of the individuals had depression score less than eight.

### Association between SCD with Depression and different socio-demographic factors

About 62.7% of the SCD with Depression subjects were females whereas 37.3% were males. Approximately 65.7% of SCD with Depression individuals were single. Most of the SCD with Depression subjects 91% were originally from Jizan and Mahayel Aseer. Furthermore 83.6% of the SCD with Depression group were educated and 59.7% had average to poor monthly income. Around 62.7% of the SCD with Depression individuals were not working, on the other hand 35.8% were students. Overall, socio-demographic factors were not significant predictors (p>0.05) of depression in these patients. (Table-II)

**Table-II:** Association between SCD with Depression and different socio-demographic factors.

Variables	≥ 8 Depressed N=67	< 8 Non-depressed N=11	P value
Age (years) Mean ± (SD) = 2	26.2±8.7	28±12.2	< 0.54
***Gender***			
Males	25(37.3)	3(27.3)	< 0.52
Females	42(62.7)	8(72.7)	
***Marital Status***			
Single	44(65.7)	7(63.6)	
Married	22(32.8)	3(27.3)	< 0.33
Divorced or Widowed	1(1.5)	1(9.1)	
***Birthplace and Residency***			
Abha	0	1(9.1)	
Ahead Rufydah	1(1.5)	0	
Almajardah	3(4.5)	0	
Bareq	1(1.5)	0	< 0.16
Faifa	1(1.5)	0	
Jizan	35(52.2)	9	
Khamis Mushayt	0	1(9.1)	
Mahayel Aseer	26(38.8)	0	
***Educational Level***			
Illiterate and Primary	11(16.4)	2(18.2)	< 0.25
Intermediate and Secondary	30(44.8)	4(36.4)	
University and Diploma	26(38.8)	5(45.5)	
***Monthly Income***			
Good	27(40.3)	4(36.4)	<0.72
Average	37(55.2)	7(63.6)	
Poor	3(4.5)	0	
***Occupation***			
Student	24(35.8)	5(45.5)	<0.77
Working	1(1.5)	0	
Not working	42(62.7)	6(54.5)	

## DISCUSSION

The present study aimed to assess the current prevalence of depressive symptoms among adults with sickle cell disease and to identify relevant socio-demographic factors related to depressive symptoms. Even if the sample was selected for absence of acute illness and history of abusing substances, the results of this study concur with previous studies. As with most chronic diseases, the prevalence of depressive symptoms is evident,^11^,^17^-^19^ however, it is high in our study compared to that reported in most of the previous studies.^8^,^20^

The current study did not show significant association between depressive symptoms and socio-demographic factors which was the second goal of the study, in contrast to other studies which found to be significant among patients with low income, poor social support, less than high school education and low family income, and unemployed.^8^,^12^,^20^

Our findings are better to be interpreted in the light of the following limitations. First, SCD participants were sampled from those attending out-patient services, which exclude those who do not routinely attend out-patient care either due to many reasons including transport, financial constraints, abusing alcohol or any kind of prescribed or non-prescribed drugs, or severity of illness. Second, this study was conducted at a single center and may not be reflective of patient characteristics at other institutes. Despite this, we note that this is the first study from Southern Saudi Arabia and second study from our kingdom to investigate depression in adult with SCD.

In conclusion, depression is common in adult patients with SCD. Contrary, socio-demographic factors were not significant predictors of depression in SCD patients. Our findings confirmed previous studies examining the occurrence of depression in adults with sickle cell disease. Further studies focusing on the effectiveness of different preventive strategies and the need for liaison care are recommended to improve the quality of life and probably modify the disease course at the national level.
